# Soluble HER2 promotes chronic antigen-mediated CD8⁺ T-cell dysfunction and limits immunotherapy response in HER2-low triple-negative breast cancer

**DOI:** 10.1038/s41419-026-09094-1

**Published:** 2026-07-06

**Authors:** Lanyu Jing, Zhiping Tan, Ziling Wang, Biao Xu, Yunjian Zhang, Nan Shao, Anze Yu, Ying Lin

**Affiliations:** 1 Breast Disease Center, First Affiliated Hospital, Sun Yat-sen University, Guangzhou, China; 2 Breast Department, Guangdong Provincial Hospital of Chinese Medicine, The Second Affiliated Hospital of Guangzhou University of Chinese Medicine, The Second Clinical College of Guangzhou University of Chinese Medicine, Guangdong Provincial Academy of Chinese Medical Sciences, Guangzhou, China; 3 Department of Urology, First Affiliated Hospital, Sun Yat-sen University, Guangzhou, China

**Keywords:** Breast cancer, Predictive markers

## Abstract

HER2-low triple-negative breast cancer (TNBC) has emerged as a clinically relevant subgroup with distinct therapeutic implications. However, the biological mechanisms underlying its heterogeneous response to immunotherapy remain poorly defined. In this manuscript, we conducted a retrospective translational analysis of patients with HER2-zero and HER2-low TNBC. Peripheral blood mononuclear cells (PBMCs) were analyzed by flow cytometry to characterize CD8⁺ T-cell differentiation and functional states. Circulating levels of soluble HER2 extracellular domain (HER2 ECD) were quantified by ELISA, and serum cytokine profiles were assessed using Luminex assays. In vitro assays using tumor-derived conditioned media and purified recombinant HER2 ECD were performed to evaluate the immunomodulatory effects of HER2 antigen exposure. Clinical responses to neoadjuvant chemotherapy with or without PD-1-based immunotherapy were compared between subgroups. In vivo validation was conducted using a syngeneic mouse model. Although the overall proportions of peripheral T cells were comparable between groups, HER2-low TNBC was associated with a shift in CD8⁺ T-cell differentiation toward effector and effector-memory phenotypes, accompanied by increased expression of exhaustion markers. Patients with HER2-low disease exhibited significantly elevated circulating HER2 ECD levels. In vitro, purified recombinant HER2 ECD was sufficient to induce time-dependent activation and subsequent exhaustion-like phenotypes in CD8⁺ T cells, while HER2 blockade in conditioned media partially attenuated these effects. Clinically, HER2-low tumors showed a significantly reduced pathological complete response rate following PD-1-based immunotherapy. Consistently, HER2-targeted therapy enhanced antitumor efficacy and improved CD8⁺ T-cell function when combined with PD-1 blockade in vivo. In summary, HER2-low TNBC is associated with elevated circulating HER2 antigen and a peripheral CD8⁺ T-cell state characterized by progressive differentiation and exhaustion. These findings support a model in which tumor antigen shedding contributes to systemic immune modulation and may limit responsiveness to immunotherapy. Targeting HER2-associated antigen dynamics may represent a potential strategy to improve immunotherapeutic outcomes in HER2-low TNBC.

## Introduction

Breast cancer is a biologically heterogeneous disease comprising multiple clinically distinct subtypes with divergent therapeutic strategies and outcomes [1–3]. In current clinical practice, classification is primarily based on immunohistochemical (IHC) assessment of estrogen receptor (ER), progesterone receptor (PgR), human epidermal growth factor receptor 2 (HER2), and Ki67 [4–7]. HER2 status is determined by IHC and in situ hybridization (ISH), with tumors defined as HER2-positive when exhibiting IHC 3+ or IHC 2+/ISH+, while all others are categorized as HER2-negative [8–11]. Notably, a substantial proportion of tumors within the HER2-negative category display low levels of HER2 expression (IHC 1+ or IHC 2+/ISH–), now referred to as HER2-low breast cancer [12, 13]. This subgroup accounts for more than half of conventionally defined HER2-negative cases, underscoring its clinical relevance and the need for refined biological characterization [13–15].

Although categorized as HER2-negative, HER2-low breast cancers retain detectable HER2 protein expression and exhibit distinct biological and therapeutic characteristics [15–19]. Despite weaker HER2-driven signaling compared with HER2-overexpressing tumors, this subgroup has demonstrated sensitivity to novel HER2-targeted agents, particularly antibody drug conjugates [8, 9]. The DESTINY-Breast04 trial established trastuzumab deruxtecan, also known as T-DXd or DS-8201, as an effective treatment for advanced HER2-low breast cancer, with notable clinical benefit observed in the hormone receptor-negative population [17, 18]. Within hormone receptor-negative disease, HER2-low and HER2-zero tumors, both commonly classified as triple-negative breast cancer, represent biologically distinct entities. Emerging evidence indicates that these subgroups differ in genomic features, tumor immune microenvironment, and therapeutic responsiveness [20, 21]. These findings highlight the need to further delineate the biological basis underlying HER2-low triple-negative breast cancer, particularly in the context of immune regulation and response to emerging therapies.

Triple-negative breast cancer is characterized by high aggressiveness and marked heterogeneity [2, 22]. In the absence of well-defined therapeutic targets, chemotherapy remains the cornerstone of treatment. In recent years, immune checkpoint inhibitors such as pembrolizumab have expanded the therapeutic landscape [2, 23, 24]. Clinical trials including KEYNOTE-522 and KEYNOTE-355 have demonstrated that immunotherapy improves survival in advanced disease and increases pathological complete response rates in the neoadjuvant setting, supporting its clinical benefit in this population [25–27]. However, therapeutic responses remain highly variable, and a substantial proportion of patients derive limited benefit from immunotherapy. These observations highlight the presence of underlying biological heterogeneity that governs treatment sensitivity, yet the mechanisms driving differential immune responses in triple-negative breast cancer remain incompletely understood.

Recent conceptual frameworks of antitumor immunity emphasize that effective immune responses depend not only on T-cell activation but also on the systemic immune context in which antigen exposure occurs [28]. Peripheral immune states may shape T-cell differentiation prior to tumor infiltration, thereby influencing subsequent tumor-directed responses [29]. In this context, circulating tumor-associated antigens represent a potential driver of systemic immune modulation, capable of priming T cells toward distinct functional states before they encounter the tumor microenvironment. However, whether such systemic regulation contributes to immune heterogeneity in clinically defined subgroups such as HER2-low triple-negative breast cancer remains unclear. Emerging evidence suggests that HER2-low triple-negative breast cancer may exhibit distinct immunological features compared with HER2-zero disease, yet the underlying mechanisms remain poorly defined [30, 31]. In particular, differences in peripheral immune states and T-cell functional programs may contribute to divergent responses to immunotherapy. These considerations raise the possibility that HER2-associated antigen exposure could influence systemic T-cell differentiation and thereby shape therapeutic outcomes, although this hypothesis has not been directly investigated.

In this study, we systematically investigated immunological differences between HER2-low and HER2-zero triple-negative breast cancer, with a particular focus on peripheral CD8⁺ T-cell phenotypes and their clinical relevance. By analyzing peripheral blood mononuclear cells from patients receiving neoadjuvant therapy, we characterized T-cell composition and differentiation states in relation to treatment exposure. In parallel, we quantified circulating HER2 antigen levels and examined their association with systemic immune features. To further explore potential mechanisms, we integrated in vitro assays and in vivo models to evaluate the impact of HER2-associated antigen exposure on T-cell functional states. Through this translational approach, we aimed to delineate a link between tumor-derived antigen dynamics and peripheral immune modulation, thereby providing biological insight into the heterogeneous immunotherapy responses observed in HER2-low triple-negative breast cancer.

## Results

### Patient characteristics

**Fig. 1:**
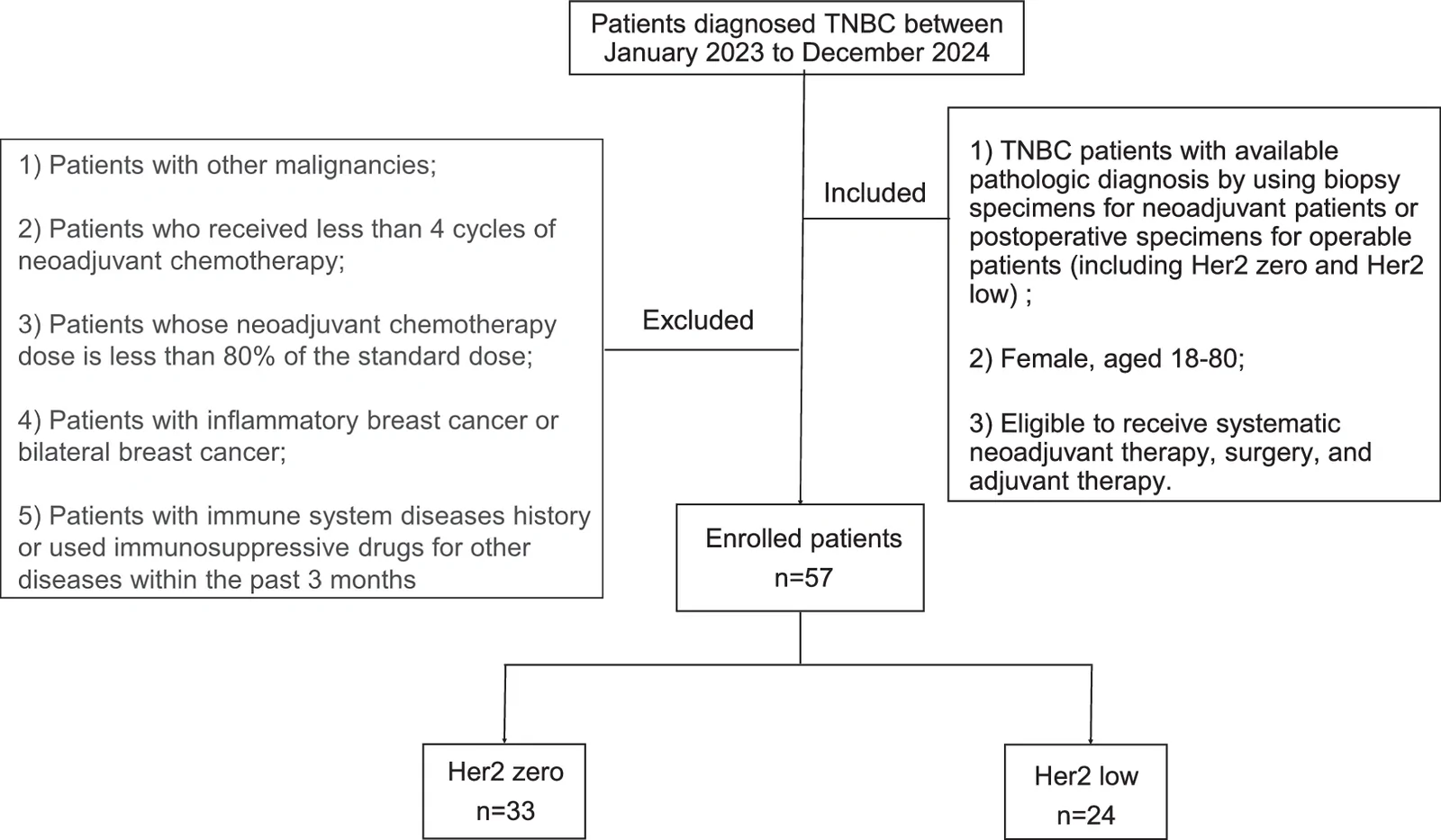
Patient selection and study design.

A total of 57 patients with triple-negative breast cancer were retrospectively enrolled from the First Affiliated Hospital of Sun Yat-sen University and Guangdong Provincial Hospital of Chinese Medicine between January 2023 and December 2024. The patient selection process is illustrated in Fig. 1. Eligible patients were female, aged 18–80 years, with pathologically confirmed triple-negative breast cancer and complete clinical data. All patients were candidates for systemic neoadjuvant therapy followed by surgery and adjuvant treatment. Patients with other malignancies, inflammatory or bilateral breast cancer, autoimmune diseases, recent immunosuppressive treatment, or inadequate neoadjuvant chemotherapy exposure, defined as fewer than four cycles or less than 80% of the standard dose intensity, were excluded. Based on HER2 expression status determined by immunohistochemistry, patients were classified into a HER2-zero group (*n* = 33) and a HER2-low group (*n* = 24). Baseline clinicopathological characteristics are summarized in Supplementary Table 1. No significant differences were observed between the two groups with respect to age, menopausal status, TNM stage, or Ki67 index, indicating comparable baseline characteristics.

### Comparable proportions of major peripheral T-cell subsets in HER2-low and HER2-zero TNBC

**Fig. 2:**
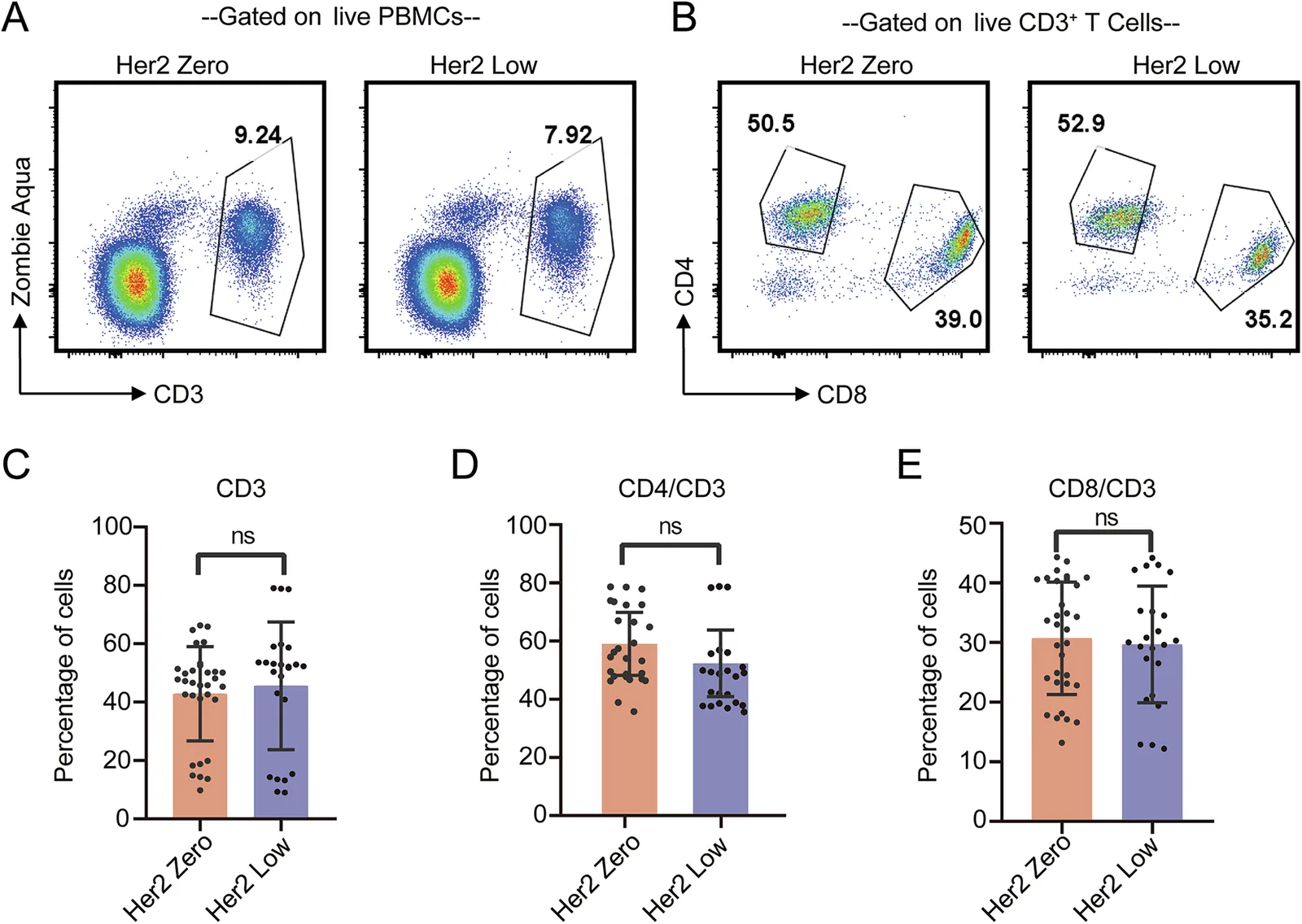
Comparable proportions of major peripheral T-cell subsets in HER2-low and HER2-zero TNBC. **A** Gating strategy for CD3^+^ T cells in peripheral blood. **B** Gating strategy for CD4^+^ and CD8^+^ T-cell subsets. **C**–**E** Quantification of CD3^+^ (**C**), CD4^+^ (**D**), and CD8^+^ (**E**) T-cell proportions in peripheral blood of HER2-low and HER2-zero TNBC patients. No statistically significant differences were observed.

We first evaluated the distribution of major peripheral T-cell subsets in patients with HER2-low and HER2-zero triple-negative breast cancer. Flow cytometry analysis showed that the proportions of CD3⁺ T cells (Fig. 2A), CD4⁺ T cells, and CD8⁺ T cells (Fig. 2B) were comparable between the two groups. Consistently, quantitative analysis confirmed no significant differences in the frequencies of CD3⁺ T cells (Fig. 2C), CD4⁺ T cells (Fig. 2D), or CD8⁺ T cells (Fig. 2E) between HER2-low and HER2-zero patients.

### Altered differentiation toward an antigen-experienced, activation–exhaustion hybrid state in peripheral CD8+ T cells associated with HER2-low TNBC

**Fig. 3:**
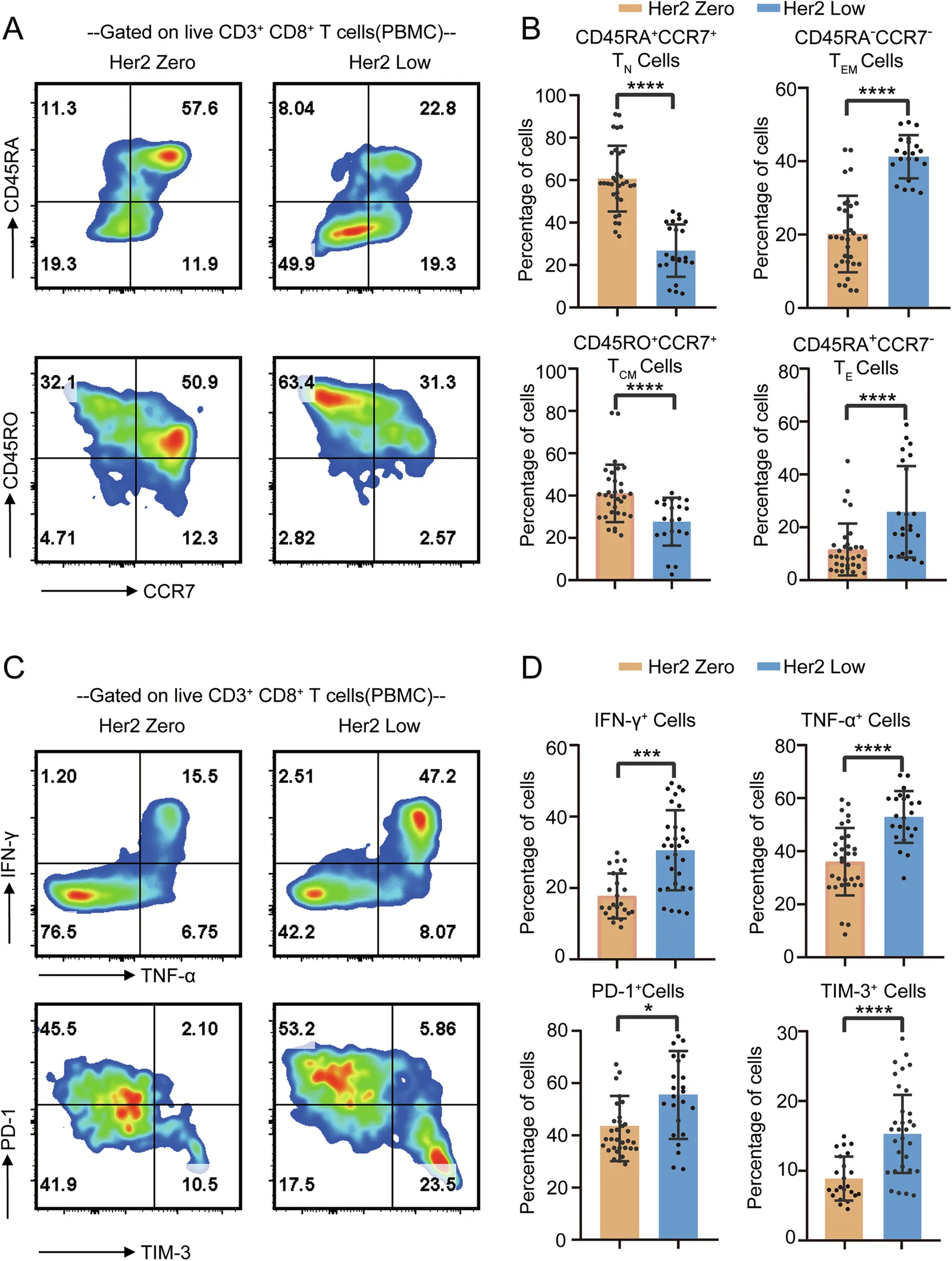
Altered differentiation toward an activated yet exhaustion-prone state in peripheral CD8+ T cells associated with HER2-low TNBC. **A** Representative flow cytometry plots of peripheral CD8⁺ T-cell subsets (gated on live CD3⁺CD8⁺ PBMCs) based on CD45RA and CCR7 expression in HER2-zero and HER2-low patients. **B** Quantification of naive (CD45RA⁺CCR7⁺), central memory (CD45RO⁺CCR7⁺), effector memory (CD45RA^−^CCR7^−^), and effector (CD45RA⁺CCR7^−^) CD8⁺ T-cell subsets in HER2-low versus HER2-zero patients. **C** Representative flow cytometry plots showing functional and exhaustion marker expression (IFN-γ, TNF-α, TIM-3, and PD-1) in peripheral CD8⁺ T cells. **D** Quantification of IFN-γ⁺, TNF-α⁺, TIM-3⁺, and PD-1⁺ CD8⁺ T cells in HER2-low versus HER2-zero patients. Data are presented as mean ± SEM. *P < 0.05, **P < 0.01, ***P < 0.001, ****P < 0.0001.

We next examined the differentiation and functional states of peripheral CD8⁺ T cells in patients with HER2-low and HER2-zero triple-negative breast cancer. Flow cytometry analysis revealed a marked shift in T-cell differentiation associated with HER2 status. Compared with HER2-low patients, those with HER2-zero disease exhibited higher proportions of CD45RA⁺CCR7⁺ naive and CD45RO⁺CCR7⁺ central memory CD8⁺ T cells, whereas the frequencies of CD45RA⁻CCR7⁻ effector memory and CD45RA⁺CCR7⁻ effector CD8⁺ T cells were significantly reduced (Fig. 3A, B). We further evaluated the functional characteristics of peripheral CD8⁺ T cells. HER2-low patients displayed increased frequencies of IFN-γ⁺ and TNF-α⁺ CD8⁺ T cells compared with HER2-zero patients (Fig. 3C, D), indicating enhanced effector-associated cytokine production. Notably, HER2-low disease was also associated with elevated expression of inhibitory receptors, with significantly higher proportions of PD-1⁺ and TIM-3⁺ CD8⁺ T cells (Fig. 3C, D). Collectively, these findings indicate that HER2-low triple-negative breast cancer is associated with a peripheral CD8⁺ T-cell state characterized by enhanced differentiation toward effector phenotypes together with increased expression of exhaustion-associated markers.

### Impaired functional reinvigoration and enhanced exhaustion of peripheral CD8+ T cells in HER2-low TNBC patients receiving PD-1-based immunotherapy

**Fig. 4:**
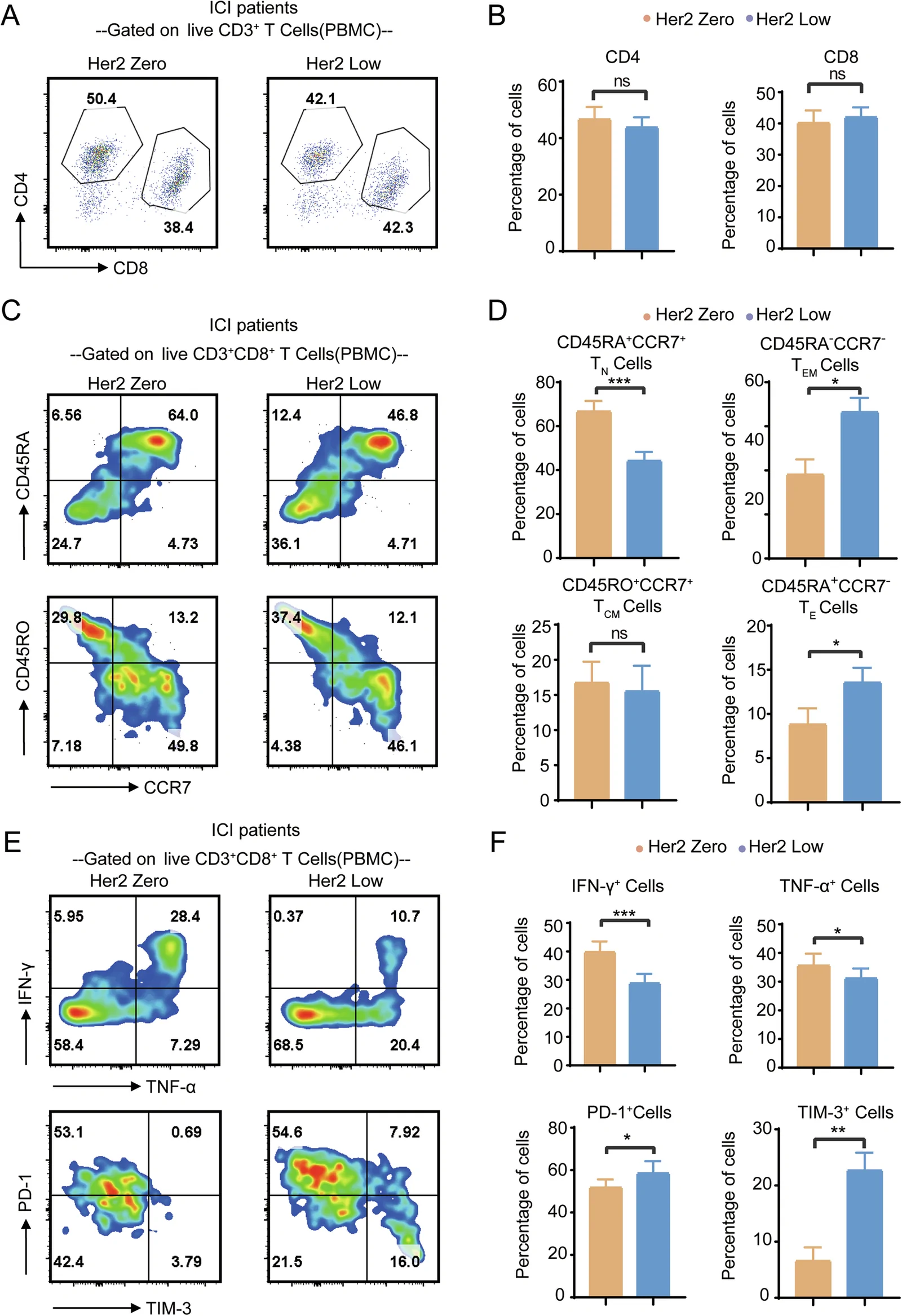
HER2-low status is associated with an activated yet functionally impaired peripheral CD8+ T-cell profile in ICI-treated TNBC patients. **A** Representative flow cytometry plot showing proportions of CD4⁺ and CD8⁺ T cells in peripheral blood (gated on live CD3⁺ T cells) from HER2-zero and HER2-low patients receiving immune checkpoint inhibitor (ICI)-based therapy. **B** Quantification of CD4⁺ and CD8⁺ T-cell proportions in HER2-low versus HER2-zero patients. No significant differences were observed. **C** Representative flow cytometry plots of peripheral CD8⁺ T-cell subsets (gated on live CD3⁺CD8⁺ PBMCs) based on CD45RA and CCR7 expression in HER2-zero and HER2-low patients treated with ICI. **D** Quantification of naive (CD45RA⁺CCR7⁺), central memory (CD45RO⁺CCR7⁺), effector memory (CD45RA^−^CCR7^−^), and effector (CD45RA⁺CCR7^−^) CD8⁺ T-cell subsets in HER2-low versus HER2-zero patients. **E** Representative flow cytometry plots showing functional and exhaustion marker expression (IFN-γ, TNF-α, TIM-3, and PD-1) in peripheral CD8⁺ T cells. **F** Quantification of IFN-γ⁺, TNF-α⁺, TIM-3⁺, and PD-1⁺ CD8⁺ T cells in HER2-low versus HER2-zero patients. Data are presented as mean ± SEM. ns, not significant; *P < 0.05, **P < 0.01, ***P < 0.001, ****P < 0.0001.

To determine whether HER2-associated immune features influence responses under immune checkpoint blockade, we analyzed peripheral T-cell characteristics in patients receiving PD-1–based therapy. The overall proportions of CD4⁺ and CD8⁺ T cells were comparable between HER2-zero and HER2-low patients (Fig. 4A, B), indicating that HER2 status does not significantly affect global T-cell abundance during immunotherapy. Consistent with observations in the overall cohort, HER2-low patients exhibited a shift toward a more differentiated CD8⁺ T-cell phenotype, characterized by reduced naive T cells and increased effector memory and effector subsets (Fig. 4C, D). However, in contrast to the baseline functional profile, HER2-low patients displayed impaired effector function in the context of immunotherapy, with significantly reduced frequencies of IFN-γ and TNF-α-producing CD8⁺ T cells compared with HER2-zero patients (Fig. 4E, F). Moreover, HER2-low patients exhibited a marked increase in exhaustion-associated markers, including TIM-3, while PD-1 expression showed no significant difference between groups (Fig. 4E, F), suggesting a shift toward a more terminally exhausted phenotype. These findings indicate that although HER2-low patients retain a differentiated, antigen-experienced CD8⁺ T-cell phenotype, this state does not translate into effective functional reinvigoration under PD-1 blockade, but is instead associated with a more exhaustion-skewed immune profile. Together, these results suggest that HER2-low TNBC is associated with impaired peripheral CD8⁺ T-cell functionality during immunotherapy, despite a pre-existing differentiated phenotype.

### HER2-derived soluble antigen induces progressive activation and exhaustion of CD8+ T cells

**Fig. 5:**
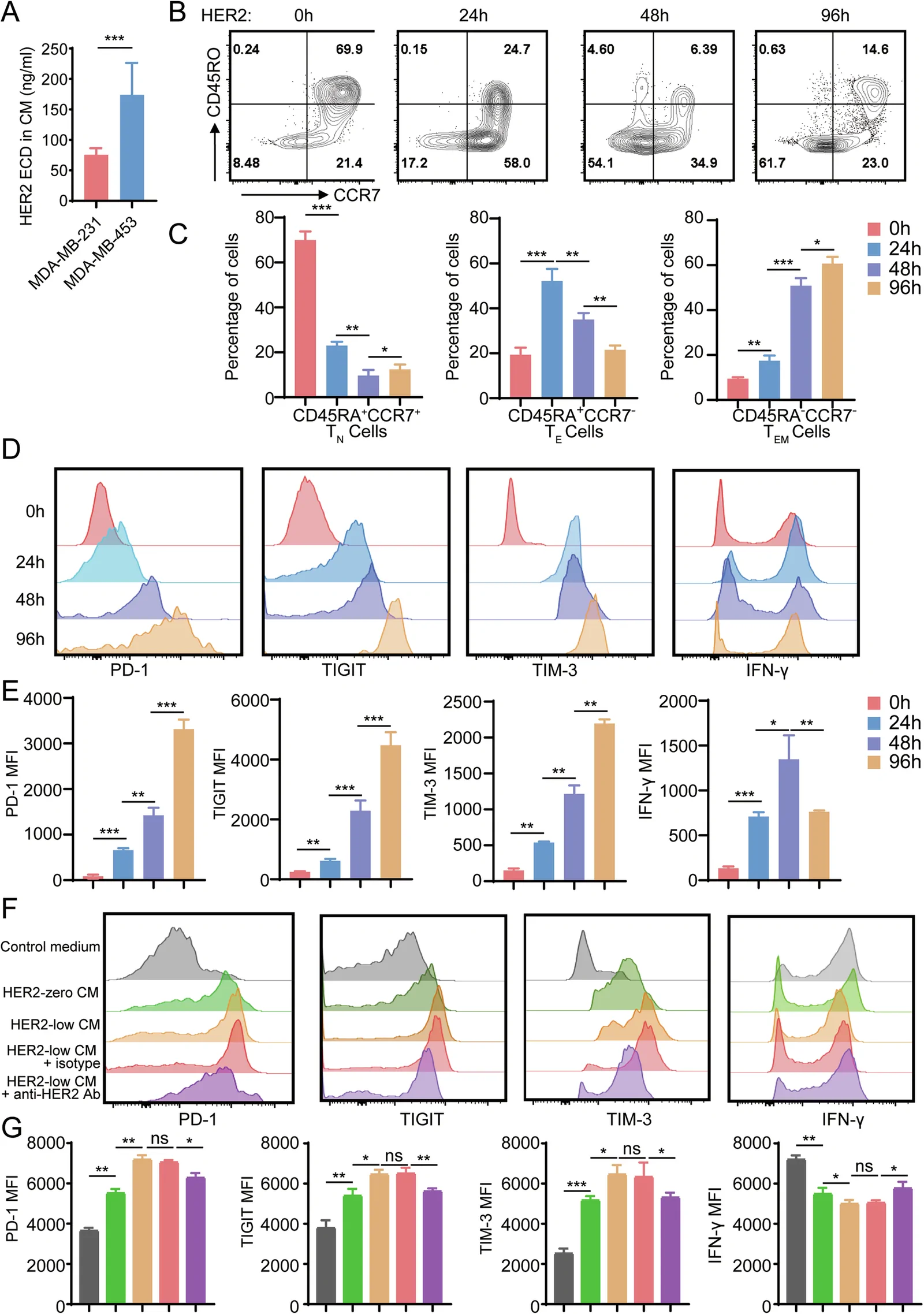
Purified recombinant HER2 extracellular domain induces progressive activation and exhaustion of CD8+ T cells and is partially reversed by HER2 blockade. **A** ELISA quantification of soluble HER2 extracellular domain (HER2 ECD) levels in conditioned medium (CM) from MDA-MB-231 and MDA-MB-453 cells. **B** Representative flow cytometry plots of CD8⁺ T-cell subsets following stimulation with purified recombinant HER2 ECD at the indicated time points (0, 24, 48, and 96 h). **C** Quantification of naive, effector, and effector memory CD8⁺ T-cell subsets over time after recombinant HER2 ECD stimulation. **D** Representative histograms showing expression of PD-1, TIGIT, TIM-3, and IFN-γ in CD8⁺ T cells after recombinant HER2 ECD stimulation. **E** Quantification of MFI of PD-1, TIGIT, TIM-3, and IFN-γ in CD8⁺ T cells over time after recombinant HER2 ECD stimulation. **F** Representative histograms showing expression of PD-1, TIGIT, TIM-3, and IFN-γ in CD8⁺ T cells cultured with control medium, HER2-zero CM, HER2-low CM, HER2-low CM with isotype control, or HER2-low CM with anti-HER2 neutralizing antibody. **G** Quantification of MFI for PD-1, TIGIT, TIM-3, and IFN-γ under the indicated conditions. The recombinant HER2 ECD stimulation assay was performed using purified CD8⁺ T cells from one healthy donor with technical replicates. Data are presented as mean ± SEM of technical replicates unless otherwise indicated. ns, not significant; *P < 0.05, **P < 0.01, ***P < 0.001.

To investigate whether HER2-associated factors contribute to the peripheral immune alterations observed in patients, we first quantified soluble HER2 extracellular domain (HER2 ECD) levels in tumor cell-conditioned media. HER2-expressing cells released significantly higher levels of HER2 ECD compared with HER2-low/negative cells (Fig. 5A), indicating that tumor-derived soluble HER2 may represent a systemic antigenic stimulus. We next assessed whether HER2 ECD could directly modulate CD8⁺ T-cell states in vitro. In a proof-of-concept in vitro assay using purified CD8⁺ T cells from one healthy donor, prolonged exposure to recombinant HER2 ECD induced a progressive shift in CD8⁺ T-cell differentiation, characterized by a marked reduction in naive cells and a corresponding increase in effector and effector memory subsets (Fig. 5B, C). This phenotype closely recapitulates the differentiation pattern observed in HER2-low patients. In parallel, CD8⁺ T cells exhibited time-dependent upregulation of activation- and exhaustion-associated markers, including PD-1, TIGIT, and TIM-3, along with dynamic changes in IFN-γ production (Fig. 5D, E), consistent with a chronic antigen stimulation-driven activation-to-exhaustion trajectory. Notably, this progressive functional remodeling mirrors the impaired effector function and exhaustion phenotype observed in HER2-low patients under immunotherapy. To further determine whether tumor-derived HER2 mediates these effects, CD8⁺ T cells were cultured with conditioned media from HER2-low tumor cells in the presence or absence of HER2-neutralizing antibody. HER2 blockade significantly attenuated the upregulation of exhaustion markers and partially restored cytokine production (Fig. 5F, G), indicating that soluble HER2 is functionally involved in driving CD8⁺ T-cell activation and exhaustion. Together with the patient-level observations, these proof-of-concept findings support a model in which tumor-derived soluble HER2 may contribute to chronic antigen-associated remodeling of peripheral CD8⁺ T cells.

### Circulating HER2 antigen is associated with systemic immune activation and T-cell differentiation in HER2-low TNBC

**Fig. 6:**
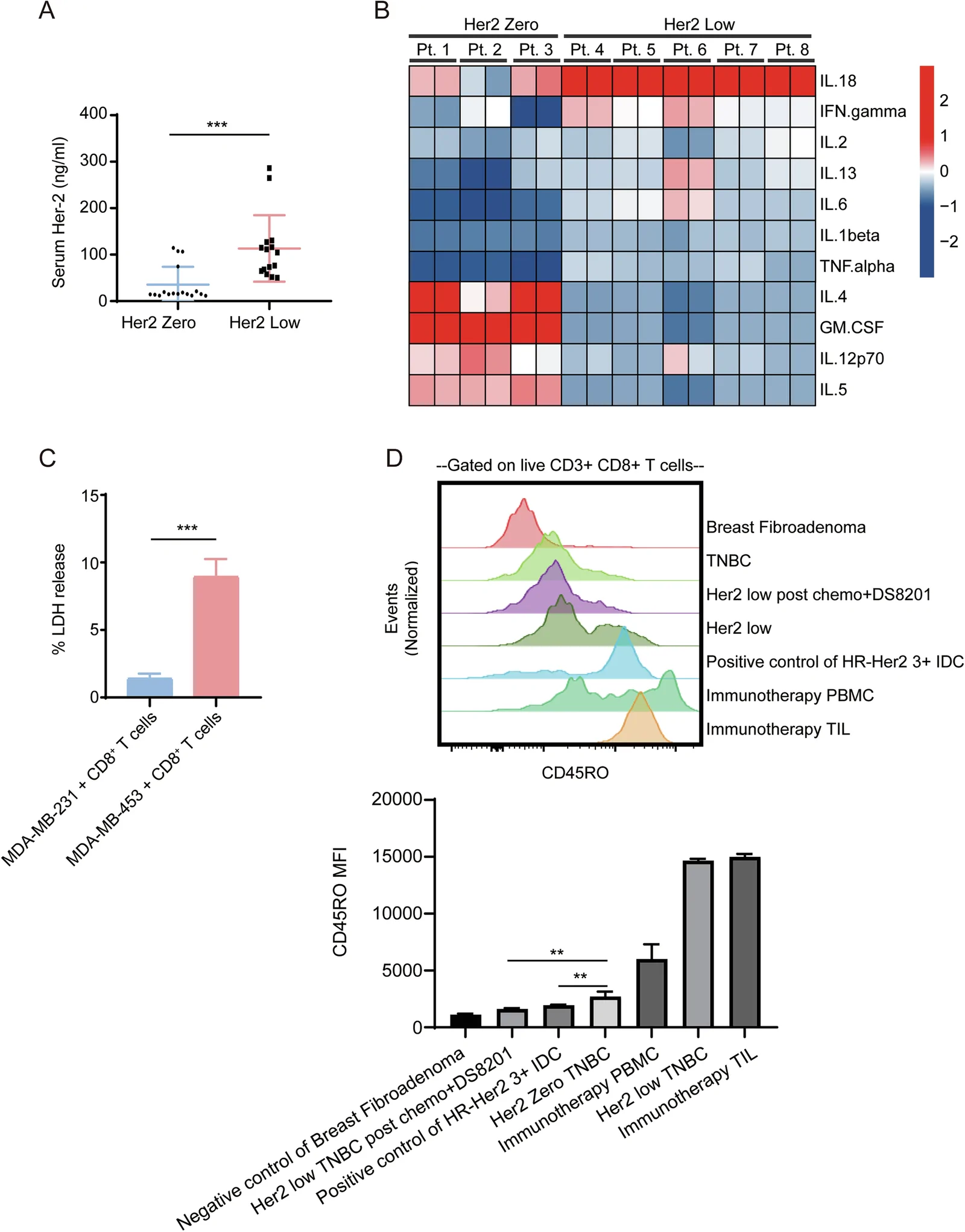
Elevated circulating HER2 antigen is associated with systemic immune activation and T-cell differentiation in HER2-low TNBC. **A** Serum levels of soluble HER2 extracellular domain (HER2 ECD) in HER2-zero and HER2-low TNBC patients measured by ELISA. **B** Heatmap showing relative cytokine levels in peripheral blood serum from HER2-zero and HER2-low patients, as measured by Luminex assay. Each column represents an individual patient, and cytokine values were calculated from repeated technical wells before visualization. **C** LDH cytotoxicity assay showing CD8⁺ T cell-mediated killing of MDA-MB-231 and MDA-MB-453 cells. **D** Representative histograms and quantification of CD45RO expression and mean fluorescence intensity in CD8⁺ T cells from different clinical groups. Fibroadenoma samples were included as a non-malignant comparator, HER2-positive breast cancer samples as a high HER2 antigen exposure reference, and post DS8201 samples as an exploratory post HER2-targeted treatment context. These groups were used to aid the interpretation of CD45RO expression across different HER2-related antigen exposure conditions. Data are presented as mean ± SEM. **P < 0.01, ***P < 0.001.

To validate the clinical relevance of HER2-driven immune modulation, we quantified circulating HER2 extracellular domain (HER2 ECD) levels in patient serum. HER2-low patients exhibited significantly higher levels of serum HER2 compared with HER2-zero patients (Fig. 6A), supporting the presence of a systemic source of soluble HER2 antigen in vivo. We next profiled circulating cytokines to assess systemic immune activation. HER2-low patients displayed a distinct inflammatory cytokine pattern, characterized by elevated levels of multiple cytokines (Fig. 6B). In this heatmap, each column represents an individual patient, and cytokine values for each patient were calculated from repeated technical wells before visualization. The elevated cytokine pattern in the HER2-low group was observed across multiple patients rather than being driven by a single extreme outlier, although interpatient heterogeneity was present. These findings indicate a chronically stimulated immune environment rather than an acute immune response. To assess the immunogenic potential of HER2, we performed cytotoxicity assays using CD8⁺ T cells. HER2-expressing tumor cells induced higher levels of T cell-mediated cytotoxicity compared with HER2-negative cells (Fig. 6C), indicating that HER2 can serve as a targetable antigen capable of eliciting T-cell responses. We further evaluated CD45RO expression as a marker of antigen-experienced T cells across different clinical contexts. Fibroadenoma samples were included as a non-malignant breast disease comparator, HER2-positive breast cancer samples served as a high HER2 antigen exposure reference, and post DS8201 samples were included to provide an exploratory context after HER2-targeted therapy. These comparator groups were used to guide the interpretation of CD45RO expression under different HER2-related antigen exposure conditions, rather than to support definitive clinical comparisons among disease entities. CD8⁺ T cells from HER2-low patients exhibited higher CD45RO expression compared with HER2-zero patients, and this effect was further amplified in patients receiving immunotherapy (Fig. 6D). This pattern is consistent with persistent antigen exposure driving T-cell differentiation toward a memory/effector-like state. Collectively, these clinical findings support a model in which elevated circulating HER2 antigen in HER2-low TNBC is associated with systemic immune activation and sustained antigen exposure, which may contribute to the differentiation and functional remodeling of peripheral CD8⁺ T cells.

### HER2-targeted therapy reduces circulating HER2 antigen and enhances antitumor immunity in combination with PD-1 blockade

**Fig. 7:**
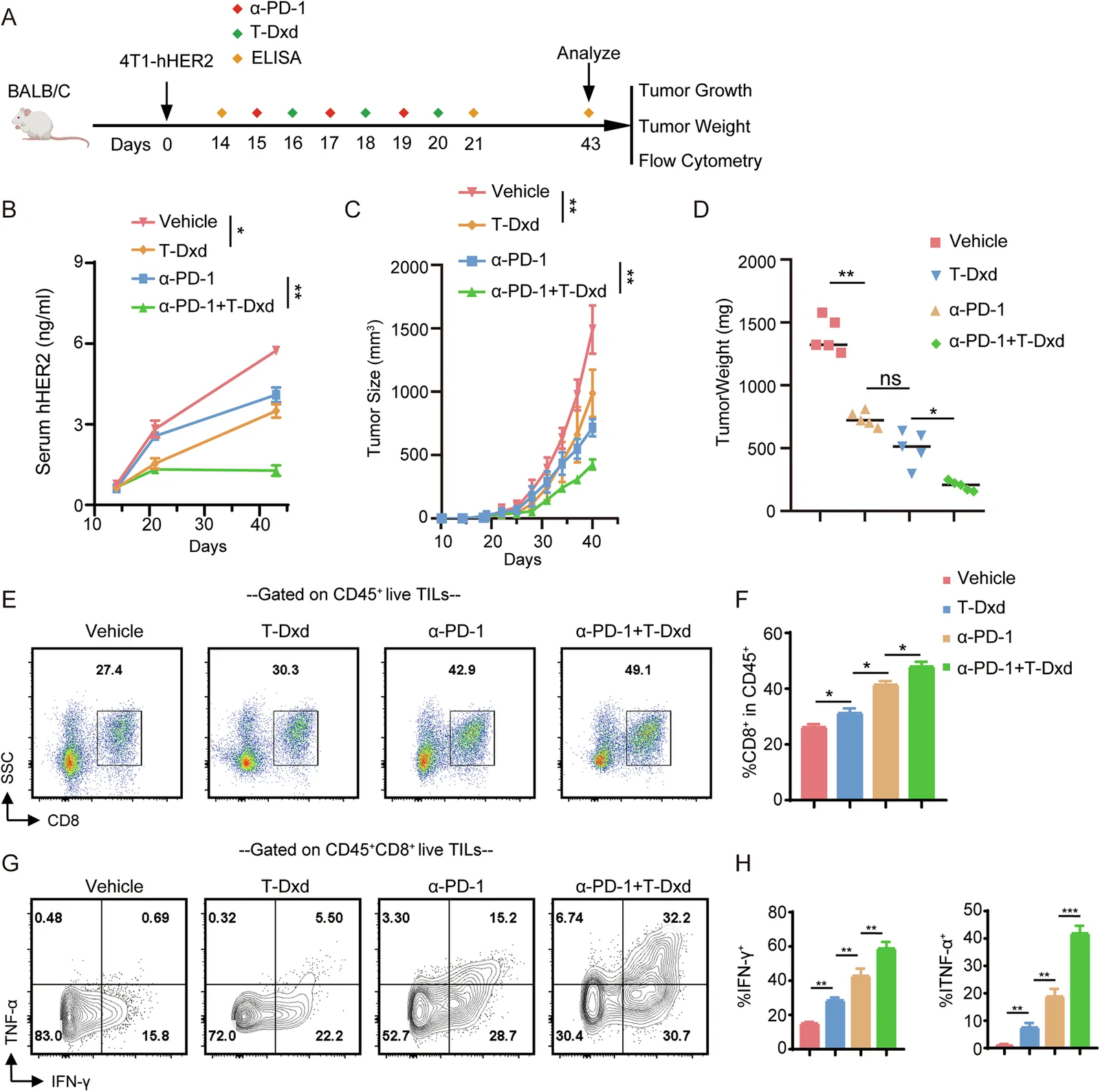
Targeting HER2 in combination with PD-1 blockade reduces circulating HER2 antigen and enhances antitumor CD8+ T-cell responses. **A** Schematic illustration of the 4T1-hHER2 syngeneic mouse model and treatment schedule. BALB/c mice were inoculated with 4T1-hHER2 tumor cells and treated with T-Dxd, anti-PD-1 antibody, or the combination. Tumor growth, serum HER2 levels, tumor weight, and immune profiling were analyzed at the indicated time points. **B** Serum levels of human HER2 extracellular domain (hHER2 ECD) measured by ELISA in different treatment groups over time. **C** Tumor growth curves of mice receiving vehicle, T-Dxd, anti-PD-1, or combination therapy. **D** Tumor weights at endpoint across treatment groups. **E** Representative flow cytometry plots showing tumor-infiltrating CD8⁺ T cells (gated on CD45⁺ live TILs) in each treatment group. **F** Quantification of CD8⁺ T-cell infiltration (% of CD45⁺ cells) in tumors. **G** Representative flow cytometry plots showing IFN-γ and TNF-α expression in tumor-infiltrating CD8⁺ T cells (gated on CD45⁺CD8⁺ live TILs). **H** Quantification of IFN-γ⁺ and TNF-α⁺ CD8⁺ T cells in tumors across treatment groups. Data are presented as mean ± SEM. ns, not significant; *P < 0.05, **P < 0.01, ***P < 0.001.

To explore the therapeutic relevance of HER2-driven immune modulation, we established a 4T1-hHER2 syngeneic mouse model and evaluated the effects of HER2-targeted therapy in combination with PD-1 blockade (Fig. 7A). Treatment with T-DXd significantly reduced circulating HER2 levels compared with vehicle control, indicating effective targeting of tumor-derived HER2 antigen in vivo (Fig. 7B). Notably, this reduction in circulating HER2 was associated with improved therapeutic responses when combined with PD-1 blockade. While both T-DXd and anti-PD-1 monotherapies modestly inhibited tumor growth, the combination treatment resulted in a markedly enhanced antitumor effect, as evidenced by reduced tumor size and tumor weight (Fig. 7C, D). Flow cytometric analysis revealed that combination therapy significantly increased the infiltration of CD8⁺ T cells within the tumor microenvironment (Fig. 7E, F), suggesting improved immune activation at the tumor site. Moreover, tumor-infiltrating CD8⁺ T cells exhibited enhanced effector function, with increased production of IFN-γ and TNF-α (Fig. 7G, H), indicating functional reinvigoration of antitumor T-cell responses. Together, these findings demonstrate that targeting HER2 not only reduces circulating tumor-derived antigen but also enhances the efficacy of PD-1 blockade by restoring CD8⁺ T-cell infiltration and effector function. These results support a model in which HER2-targeted therapy alleviates chronic antigen-driven immune dysfunction, thereby improving responsiveness to immune checkpoint inhibition.

## Discussion

The clinical relevance of HER2-low breast cancer has become increasingly evident with the emergence of antibody–drug conjugates targeting low-level HER2 expression. However, the biological basis underlying the heterogeneous response of HER2-low triple-negative breast cancer (TNBC) to immunotherapy remains incompletely defined [17, 32]. In this study, we identify a distinct peripheral immune phenotype associated with HER2-low status and propose that this state reflects chronic antigen-driven immune conditioning. Specifically, sustained exposure to tumor-derived HER2 antigen appears to drive progressive CD8⁺ T-cell differentiation accompanied by functional exhaustion, thereby limiting effective immune reinvigoration during PD-1 blockade.

A key observation is that, despite comparable proportions of total CD3⁺, CD4⁺, and CD8⁺ T cells, HER2-low TNBC is associated with a shift in T-cell differentiation toward effector and effector memory phenotypes, accompanied by a reduction in naive and central memory populations. Such differentiation patterns are characteristic of persistent antigen exposure, where continuous stimulation drives progressive T-cell maturation but ultimately leads to functional constraint and exhaustion. Consistently, HER2-low patients exhibited increased expression of exhaustion markers alongside altered cytokine production, suggesting a state of antigen-experienced but functionally impaired immunity rather than effective antitumor activation.

Importantly, these systemic immune features were observed in parallel with elevated levels of circulating HER2 extracellular domain (ECD), consistent with previous reports of HER2 shedding in breast cancer. Emerging evidence suggests that circulating tumor-associated antigens can shape systemic immunity prior to tumor infiltration, thereby influencing downstream antitumor responses. In line with this concept, our in vitro data demonstrate that HER2 ECD is sufficient to induce time-dependent CD8⁺ T-cell differentiation and upregulation of exhaustion-associated markers, recapitulating the phenotype observed in patients. Furthermore, HER2 blockade partially attenuated these effects, supporting a functional role of soluble HER2 in driving peripheral immune remodeling. The partial rather than complete reversal observed after HER2 blockade suggests that additional tumor-derived factors in conditioned media, such as cytokines, metabolites, extracellular vesicle-associated signals, or other immunomodulatory components, may also contribute to CD8⁺ T-cell dysfunction. Thus, soluble HER2 may represent an important contributor to this process rather than the sole determinant of the observed phenotype. The precise molecular mechanisms by which soluble HER2 ECD modulates CD8⁺ T-cell function remain to be elucidated and may involve antigen-specific or receptor-mediated pathways. Together with the clinical observation of elevated serum HER2 and altered cytokine profiles, these findings suggest that HER2-low TNBC is characterized by a systemic, antigen-driven immune conditioning process. From a clinical perspective, HER2-low TNBC patients exhibited significantly lower pathological complete response rates following PD-1–based neoadjuvant immunotherapy compared with HER2-zero patients, whereas such differences were less pronounced in patients treated with chemotherapy alone. These findings are consistent with emerging evidence suggesting differential sensitivity to immunotherapy between HER2-low and HER2-zero TNBC. Mechanistically, our data suggest that chronic antigen-driven immune conditioning may constrain CD8⁺ T-cell reinvigoration upon PD-1 blockade.

These findings also provide a biological context for ongoing clinical strategies combining HER2-targeted therapy with immunotherapy. Trastuzumab-based agents have been shown to inhibit HER2 ECD shedding, raising the possibility that modulation of circulating antigen levels may influence systemic immune states. In this context, antibody–drug conjugates such as trastuzumab deruxtecan may exert immunomodulatory effects beyond direct cytotoxicity [12, 13]. Notably, our in vivo data demonstrate that HER2-targeted therapy reduces circulating HER2 levels and, when combined with PD-1 blockade, enhances antitumor efficacy, increases CD8⁺ T-cell infiltration, and restores effector function. These findings suggest that PD-1 blockade alone may be insufficient to fully reverse the exhaustion-skewed peripheral CD8⁺ T-cell state associated with HER2-low TNBC, whereas reducing HER2-associated antigenic pressure through HER2-targeted therapy may facilitate more effective T-cell reinvigoration during PD-1 blockade. Nevertheless, peripheral CD8⁺ T-cell states may not fully mirror intratumoral T-cell phenotypes, which are additionally shaped by local antigen presentation, cytokine signaling, stromal organization, and suppressive immune components. Future studies using matched peripheral and tumor samples will be needed to clarify the functional relationship between systemic and intratumoral T-cell states.

This study has several limitations. The retrospective design and modest cohort size, particularly within the immunotherapy-treated subgroup, limit the statistical power of clinical comparisons. In addition, our analysis primarily focused on peripheral immune features, while comprehensive characterization of intratumoral immune composition, spatial organization, and tumor-draining lymph nodes was beyond the scope of this study. Future prospective studies integrating systemic and intratumoral immune profiling will be required to further elucidate the interplay between HER2-associated antigen dynamics and antitumor immunity. In addition, the recombinant HER2 ECD stimulation assay was performed using CD8⁺ T cells from a single healthy donor with technical replicates. Therefore, this experiment should be interpreted as proof-of-concept evidence showing that HER2 ECD can induce CD8⁺ T-cell phenotypic remodeling under controlled in vitro conditions. Future studies using multiple independent donors will be needed to evaluate donor-to-donor variability and confirm the generalizability of this response.

In summary, our study identifies a distinct peripheral immune state associated with HER2-low TNBC and provides evidence that circulating HER2 antigen may act as a chronic immune stimulus shaping systemic CD8⁺ T-cell differentiation and exhaustion. We propose a model in which sustained exposure to soluble HER2 drives antigen-experienced but functionally constrained T-cell states, limiting effective reinvigoration during PD-1 blockade, while HER2-targeted therapy can partially reverse this process. These findings provide new insight into the biological heterogeneity of HER2-low disease and support the development of rational combination strategies integrating HER2-targeted therapy with immune checkpoint inhibition.

## Materials and methods

### Patients and clinical samples

This study retrospectively enrolled a total of 57 patients with triple-negative breast cancer (TNBC) from the First Affiliated Hospital of Sun Yat-sen University and Guangdong Provincial Hospital of Chinese Medicine between January 2023 and December 2024. The patient inclusion and exclusion criteria are detailed in the flow chart (Fig. 1). Briefly, eligible patients were females aged 18 to 80 years with a confirmed pathological diagnosis of TNBC, defined as estrogen receptor (ER) and progesterone receptor (PR) expression in <1% of tumor cells, and HER2-negative status (IHC 0 or 1+/2+ with negative FISH).

Patients were categorized into HER2-zero (IHC 0, *n* = 33) and HER2-low (IHC 1+ or IHC 2 + /FISH–, *n* = 24) groups according to the American Society of Clinical Oncology/College of American Pathologists (ASCO/CAP) guidelines. Peripheral blood samples were collected before the initiation of systemic neoadjuvant therapy for baseline immune profiling. For patients receiving PD-1-based neoadjuvant therapy, blood samples used for the on-treatment immune analysis were collected before the next treatment cycle and at least 14 days after the previous administration of chemotherapy or PD-1 antibody, when clinically feasible. Patients receiving systemic corticosteroids, immunosuppressive agents, or other immune-modulating medications within X weeks before blood collection were excluded. PBMCs and serum were isolated within 2 h of blood collection and processed for downstream analyses. All patients provided written informed consent, and the study was approved by the Ethics Committees of the participating institutions.

### ELISA

Serum levels of soluble HER2 extracellular domain (HER2 ECD) were measured using a human HER2 ELISA kit (Cat: JM-03471H2, JINGMEI) according to the manufacturer’s instructions. Briefly, serum samples were added to pre-coated plates and incubated to allow binding of the HER2 antigen to capture antibodies. After washing, enzyme-conjugated detection antibodies were added, followed by substrate solution. Absorbance was measured at 450 nm using a microplate reader. HER2 concentrations were calculated based on standard curves.

### PBMC isolation

Peripheral blood mononuclear cells (PBMCs) were isolated using density gradient centrifugation with Lymphoprep™ (07801, STEMCELL). Briefly, whole blood was diluted with an equal volume of PBS and layered onto Lymphoprep solution, followed by centrifugation at 800×*g* for 20 min at room temperature. The PBMC layer was collected, washed twice with PBS, and resuspended for subsequent experiments.

### Luminex xMAP

Serum cytokine levels were quantified using the 11-Plex ProcartaPlex kit (EPX110-10810-901, Invitrogen) according to the manufacturer’s protocol. Briefly, samples were incubated with antibody-coated beads, followed by detection antibodies and streptavidin-PE. Data were acquired on a Luminex platform and analyzed using dedicated software. Cytokine concentrations were calculated based on standard curves.

### Flow cytometry

PBMCs were resuspended in PBS containing 1% BSA. Cell viability was assessed using Zombie Aqua™ Fixable Viability Kit (423101, BioLegend) for 15 min at room temperature. Cells were then stained with fluorochrome-conjugated antibodies at 4 °C for 30 min in the dark. After washing, samples were analyzed using a BD LSRFortessa flow cytometer. Data were processed using FlowJo software (version 10.8.1). Doublets were excluded, and dead cells were removed prior to analysis. T-cell subsets were identified based on CD3, CD4, and CD8 expression and were further characterized using CD45RA, CD45RO, and CCR7. Within live CD3⁺CD8⁺ T cells, naive cells were defined as CD45RA⁺CCR7⁺, central memory cells as CD45RO⁺CCR7⁺, effector memory cells as CD45RA⁻CCR7⁻, and effector cells as CD45RA⁺CCR7⁻. This gating strategy was applied consistently across all relevant analyses. Functional and exhaustion markers were analyzed within gated CD8⁺ T cells.

The following antibodies were used: Pacific Blue™ anti-human CD3 (UCHT1), PerCP/Cyanine5.5 anti-human CD4 (OKT4), PE/Cyanine7 anti-human CD8 (SK1), PE anti-human CD45RO (UCHL1), APC anti-human CCR7 (G043H7), FITC anti-human CD45RA (HI100), PE anti-human PD-1 (EH12.2H7), FITC anti-human TIM-3 (F38-2E2), APC anti-human TIGIT(A15153G), PE anti-human IFN-γ (W19227A), and APC anti-human TNF-α (MAb11) (all from BioLegend).

### Cell culture and stable cell line construction

The MDA-MB-231 and MDA-MB-453 cell lines were authenticated by STR profiling, obtained from the American Type Culture Collection (ATCC, Manassas, VA, USA), and tested for mycoplasma contamination to ensure they were mycoplasma-free. These cells were cultured in DMEM medium (Gibco) supplemented with 10% FBS. To achieve HER2 overexpression, specific plasmids were constructed, and lentiviral particles were generated using a packaging system and transfected into the target cells. After transfection, cells were selected with puromycin to ensure stable integration and expression of the lentivirus.

### Recombinant HER2 ECD stimulation assay

Purified peripheral CD8⁺ T cells from one healthy donor were cultured in the presence of purified recombinant human HER2 extracellular domain (Catalog # 10126-ER, R&D Systems Inc.) at a concentration of 0.5 μg/mL for the indicated time periods (0, 24, 48, and 96 h). Technical replicates were included for each condition and time point. After stimulation, cells were collected and analyzed by flow cytometry for differentiation, activation, and exhaustion-associated markers.

### T-cell stimulation and HER2 blockade assay

Purified peripheral CD8⁺ T cells from healthy donors were cultured with conditioned media derived from HER2-low or control tumor cells. In selected experiments, anti-HER2 neutralizing antibody or an isotype control antibody was added to the culture system. Cells were incubated for indicated time points (0–96 h) and subsequently analyzed for differentiation, activation, and exhaustion markers by flow cytometry.

### LDH assay

Cytotoxicity was assessed using an LDH release assay (Cat: 88954, C0016, Beyotime) according to the manufacturer’s instructions. Briefly, supernatants were collected after co-culture of CD8⁺ T cells with target tumor cells, and LDH activity was measured at 490 nm. Cytotoxicity was calculated relative to maximum lysis controls.

### In vivo tumor model and treatment

Female BALB/c mice (6–8 weeks old) were purchased from GemPharmatech Co., Ltd (Jiangsu, China) and maintained under specific pathogen-free (SPF) conditions. All animal procedures were approved by the Institutional Animal Care and Use Committee and performed in accordance with institutional guidelines. The murine 4T1 breast cancer cell line stably expressing human HER2 (4T1-hHER2) was used to establish a syngeneic tumor model. Mice were subcutaneously inoculated in the flank with 1 × 10⁶ 4T1-hHER2 cells suspended in 100 μL PBS. When tumors reached a volume of approximately 50–100 mm³, mice were randomly assigned to the following treatment groups: vehicle control, anti-PD-1 antibody (Selleck, A2122), trastuzumab deruxtecan (T-DXd, MedChemExpress, HY-138298), and combination therapy (T-DXd plus anti-PD-1). Anti-PD-1 antibody was administered via intraperitoneal injection at a dose of 200 μg per mouse. T-DXd was administered via intravenous injection at a dose of 5 mg/kg. Combination therapy consisted of concurrent administration of both agents. Tumor size was measured every 2–3 days using calipers, and tumor volume was calculated using the formula: V = (length × width^2^)/2. At the experimental endpoint, mice were euthanized, and tumors and peripheral blood samples were collected for further analyses. The animal experiments were approved by the Ethics Committee for Clinical Research and Animal Trials of the First Affiliated Hospital of Sun Yat-sen University, with approval number [2022]-242.

### Serum HER2 measurement

Peripheral blood was collected from mice at the indicated time points, and serum was isolated by centrifugation. Levels of human HER2 extracellular domain (hHER2 ECD) were measured using ELISA as described above.

### Flow cytometry analysis of tumor-infiltrating lymphocytes (TILs)

Tumor tissues were excised, mechanically dissociated, and enzymatically digested using collagenase and DNase to obtain single-cell suspensions. Cells were filtered through a 70-μm cell strainer and washed with PBS. For flow cytometry analysis, cells were stained with viability dye followed by surface markers, including CD45 (S18009F) and CD8 (YTS156.7.7). Intracellular cytokine staining for IFN-γ (XMG1.2) and TNF-α (MP6-XT22) was performed after stimulation and fixation/permeabilization according to standard protocols. Data were acquired using a BD LSRFortessa flow cytometer and analyzed with FlowJo software. CD8⁺ T cells were gated from live CD45⁺ tumor-infiltrating leukocytes.

### Statistical analyses

Data analysis was performed using R, version 4.0.2, and GraphPad Prism, version 8.0. Data normality was assessed before statistical testing. For comparisons between two groups, normally distributed data were analyzed using an unpaired two-tailed Student’s *t* test, whereas non-normally distributed data were analyzed using the Mann–Whitney *U* test. For comparisons among more than two groups, one-way ANOVA or the Kruskal–Wallis test was used as appropriate according to data distribution. Correlations were assessed using Pearson’s or Spearman’s correlation coefficients, depending on data distribution. Given the exploratory nature and limited sample size of the immune profiling and cytokine analyses, *P* values were not adjusted for multiple testing and were interpreted descriptively. *P* value less than 0.05 was considered statistically significant.

## Supplementary information

Supplementary Table 1

Supplementary Table 2

## Data Availability

The datasets generated and/or analyzed during the current study are not publicly available due to patient privacy and institutional restrictions, but are available from the corresponding author on reasonable request.
