# Supplementary material for: Soluble HER2 promotes chronic antigen-mediated CD8⁺ T-cell dysfunction and limits immunotherapy response in HER2-low triple-negative breast cancer
Source: Cell Death Dis. 2026 Jul 6;17(1):838. doi: 10.1038/s41419-026-09094-1 (PMC13616914; doi:10.1038/s41419-026-09094-1)
Supplement: Supplementary file 1 [file 41419_2026_9094_MOESM1_ESM.docx]

**Supplementary Table 1. Baseline characteristics of HER2-zero and HER2-low TNBC patients**

| Characteristic | HER2-zero (n=33) | HER2-low (n=24) | p-value |
| --- | --- | --- | --- |
| **Age, years** | 48.2 ± 12.4 | 48.5 ± 12.8 | 0.92 |
| **Menopausal Status, n (%)** |  |  | 0.45 |
| Premenopausal | 20 (60.6) | 12 (50.0) |  |
| Postmenopausal | 13 (39.4) | 12 (50.0) |  |
| **T Stage, n (%)** |  |  | 0.34 |
| T1 | 9 (27.3) | 6 (25.0) |  |
| T2 | 19 (57.6) | 12 (50.0) |  |
| T3 | 4 (12.1) | 4 (16.7) |  |
| T4 | 1 (3.0) | 2 (8.3) |  |
| **N Stage, n (%)** |  |  | 0.28 |
| N0 | 15 (45.5) | 8 (33.3) |  |
| N1 | 10 (30.3) | 9 (37.5) |  |
| N2 | 6 (18.2) | 5 (20.8) |  |
| N3 | 2 (6.1) | 2 (8.3) |  |
| **Ki-67 Index, Mean ± SD** | 0.57 ± 0.20 | 0.59 ± 0.22 | 0.71 |
