# Supplementary material for: Soluble HER2 promotes chronic antigen-mediated CD8⁺ T-cell dysfunction and limits immunotherapy response in HER2-low triple-negative breast cancer
Source: Cell Death Dis. 2026 Jul 6;17(1):838. doi: 10.1038/s41419-026-09094-1 (PMC13616914; doi:10.1038/s41419-026-09094-1)
Supplement: Supplementary file 2 [file 41419_2026_9094_MOESM2_ESM.docx]

**Supplementary Table 2. Pathological response in TNBC patients receiving neoadjuvant therapy**

| Treatment Group | HER2 Status | n | Total pCR | T pCR | N pCR |
| --- | --- | --- | --- | --- | --- |
| **Neoadjuvant**  **Chemo-Immunotherapy** | HER2-zero | 10 | 8 (80.0%) | 8 (80.0%) | 8 (80.0%) |
|  | HER2-low | 12 | 3 (25.0%) | 3 (25.0%) | 6 (50.0%) |
| **p-value** |  |  | 0.030 | 0.030 | 0.204 |
| **Neoadjuvant**  **Chemotherapy** | HER2-zero | 11 | 5 (45.5%) | 6 (54.5%) | 9 (81.8%) |
|  | HER2-low | 5 | 1 (20.0%) | 1 (20.0%) | 3 (60.0%) |
| **p-value** |  |  | 0.329 | 0.303 | 0.586 |

pCR was defined as ypT0N0M0; T pCR as ypT0; N pCR as ypN0. P-values were calculated using Fisher's exact test.
